# Vitamin D and Wnt3A have additive and partially overlapping modulatory effects on gene expression and phenotype in human colon fibroblasts

**DOI:** 10.1038/s41598-019-44574-9

**Published:** 2019-05-30

**Authors:** Gemma Ferrer-Mayorga, Núria Niell, Ramón Cantero, José Manuel González-Sancho, Luis del Peso, Alberto Muñoz, María Jesús Larriba

**Affiliations:** 1 Instituto de Investigaciones Biomédicas “Alberto Sols”, Consejo Superior de Investigaciones Científicas, Universidad Autónoma de Madrid, Madrid, Spain; 2 Instituto de Investigación Sanitaria Hospital Universitario La Paz, Madrid, Spain; 3 CIBERONC, Instituto de Salud Carlos III, Madrid, Spain; 4 Departamento de Bioquímica, Facultad de Medicina, Universidad Autónoma de Madrid, Madrid, Spain; 5 Servicio de Cirugía General, Hospital Universitario La Paz, Madrid, Spain; 6 CIBERES, Instituto de Salud Carlos III, Madrid, Spain; 7 Present Address: Departamento de Biología, Facultad de Ciencias, Universidad Autónoma de Madrid, Madrid, Spain

**Keywords:** Cancer microenvironment, Cell migration, Nuclear receptors, Cell growth, Transcriptomics

## Abstract

The Wnt/β-catenin signalling pathway is essential for intestinal epithelium homeostasis, but its aberrant activation is a hallmark of colorectal cancer (CRC). Several studies indicate that the bioactive vitamin D metabolite 1α,25-dihydroxyvitamin D_3_ (1,25(OH)_2_D_3_) inhibits proliferation and promotes epithelial differentiation of colon carcinoma cells in part through antagonism of the Wnt/β-catenin pathway. It is now accepted that stromal fibroblasts are crucial in healthy and pathologic intestine: pericryptal myofibroblasts are constituents of the stem cell niche and cancer-associated fibroblasts (CAFs) contribute to CRC progression. However, studies on the combined action of 1,25(OH)_2_D_3_ and Wnt factors in colon fibroblasts are lacking. Here we show by global transcriptomic studies that 1,25(OH)_2_D_3_ and Wnt3A have profound, additive, partially overlapping effects on the gene expression profile of CCD-18Co human colon myofibroblasts. Moreover, 1,25(OH)_2_D_3_ and Wnt3A inhibit CCD-18Co cell proliferation and migration, while 1,25(OH)_2_D_3_ reduces, but Wnt3A increases, their capacity to contract collagen gels (a marker of fibroblast activation). These data were largely confirmed in patient-derived primary colon normal fibroblasts and CAFs, and in fibroblasts from other origins. Our results indicate that 1,25(OH)_2_D_3_ and Wnt3A are strong regulators of colon fibroblast biology and contribute to a better knowledge of intestinal homeostasis and stromal fibroblast action in CRC.

## Introduction

The intestinal epithelium (small intestine and colorectum) is the most intensively renewing adult tissue. It undergoes rapid turnover to prevent the accumulation of DNA damage due to external factors. This process is sustained by stem cells that reside at the bottom of the intestinal crypts and generate proliferative progenitors that subsequently give rise to the specialized differentiated cells. Several signals are required to maintain the homeostasis of intestinal stem cells, among them canonical Wnt factors have a prominent role^1–3^. These Wnt proteins are provided by cellular components of the stem cell niche such as Paneth cells (only in the small intestine) and pericryptal myofibroblasts^3–5^. Extracellular canonical Wnts bind to their cell membrane receptors and promote an intracellular signalling cascade (known as the Wnt/β-catenin or canonical Wnt signalling pathway) that leads to the translocation of β-catenin to the cell nucleus. There, it binds DNA-bound transcription factors of the T-cell factor (TCF) family and acts as a transcriptional co-activator for several genes that are crucial to preserve the stemness and the undifferentiated phenotype of intestinal stem cells^6^.

Colorectal cancer (CRC) is a major health problem and one of the leading causes of cancer-related deaths worldwide^7^. The initial event in most CRCs is the aberrant activation of the Wnt/β-catenin signalling pathway due to mutations in genes encoding components of the pathway (*APC*, *AXIN2*, *CTNNB1*, *RNF43*), which promotes colon epithelial cells to acquire an undifferentiated phenotype with an elevated proliferation rate and stem cell traits^6,8,9^. In addition, it has been described that colon carcinoma cells acquire the ability to secrete canonical Wnt proteins such as Wnt3A to autocrinally achieve high activation of the Wnt/β-catenin signalling pathway and increase their own survival^10,11^. Thus, elevated expression of Wnt3A has been found in human colorectal carcinomas associated with advanced stages and worse prognosis^10,12,13^.

Recent studies have shown that tumour stroma contributes to CRC progression and, accordingly, poor prognosis CRC subtypes are characterized by high stromal content and a stromal-specific gene expression program^14–16^. Cancer-associated fibroblasts (CAFs) are one of the main cellular components of tumour stroma and are derived from normal fibroblasts (NFs) or other cell types that upon activation by signals received from carcinoma cells differentiate into CAFs. They modulate the extracellular matrix properties and secrete molecules that act on carcinoma and other cells present in the tumour microenvironment promoting tumour growth and invasion, angiogenesis and immunosuppression^17–19^.

Many epidemiological studies have shown that vitamin D deficiency is associated with high CRC risk and mortality, which suggests that vitamin D has a protective effect against this disease^20,21^. The most active vitamin D metabolite 1α,25-dihydroxyvitamin D_3_ (1,25(OH)_2_D_3_, calcitriol) is a major regulator of gene expression in humans. It acts through the vitamin D receptor (VDR), a transcription factor of the nuclear receptor superfamily that upon ligand binding activates the transcription of its target genes^22,23^. 1,25(OH)_2_D_3_ promotes epithelial differentiation and inhibits proliferation in colon carcinoma cells by several mechanisms that include antagonism of the Wnt/β-catenin signalling pathway^24–26^. We recently showed that primary cultures of human colon NFs and CAFs express *VDR* and respond to 1,25(OH)_2_D_3_. Moreover, high VDR expression in tumour stromal fibroblasts is associated with a better clinical outcome in CRC, which suggests that the antitumour role of 1,25(OH)_2_D_3_ in this disease is mediated not only by its effects on carcinoma cells, but also by its action on CAFs^27^.

Although the role of the Wnt/β-catenin signalling pathway on normal intestinal stem cells and on colon carcinoma cells is relatively well described, very few studies^28^ have focused on the action that canonical Wnt proteins present in the intestinal stem cell niche and in the CRC tumour microenvironment may have on the surrounding pericryptal myofibroblasts and CAFs. Moreover, the possible interaction between 1,25(OH)_2_D_3_ and canonical Wnt factors in fibroblasts remains unknown. Thus, we hypothesized that 1,25(OH)_2_D_3_ and canonical Wnt proteins are important modulators of fibroblast biology in healthy and tumour intestine and that an interplay between both agents may exist. To address this hypothesis, we chose Wnt3A as a representative for canonical Wnt proteins because is one of the best characterized canonical Wnts and is widely considered as the prototype for this class of ligands^9,29,30^. In addition, Wnt3A is highly expressed in human CRC^10,12,13^. Our main objective was to characterize the effect of both 1,25(OH)_2_D_3_ and Wnt3A on the gene expression program and phenotype of CCD-18Co human colon myofibroblasts. Importantly, we extended the study of 1,25(OH)_2_D_3_ and Wnt3A action to primary cultures of human colon NFs and CAFs derived from CRC patients, and to IMR-90 human lung fibroblasts and BJ-hTERT human foreskin fibroblasts. Our results indicate that 1,25(OH)_2_D_3_ and Wnt3A are crucial regulators of the gene expression and phenotype of human colon fibroblasts and may contribute to a better understanding of intestinal homeostasis and more efficient treatment of intestinal pathologies such as CRC, inflammatory bowel diseases, and intestinal fibrosis.

## Results

### 1,25(OH)_2_D_3_ and Wnt3A have additive gene regulatory effects in human colon myofibroblasts

**Figure 1:**
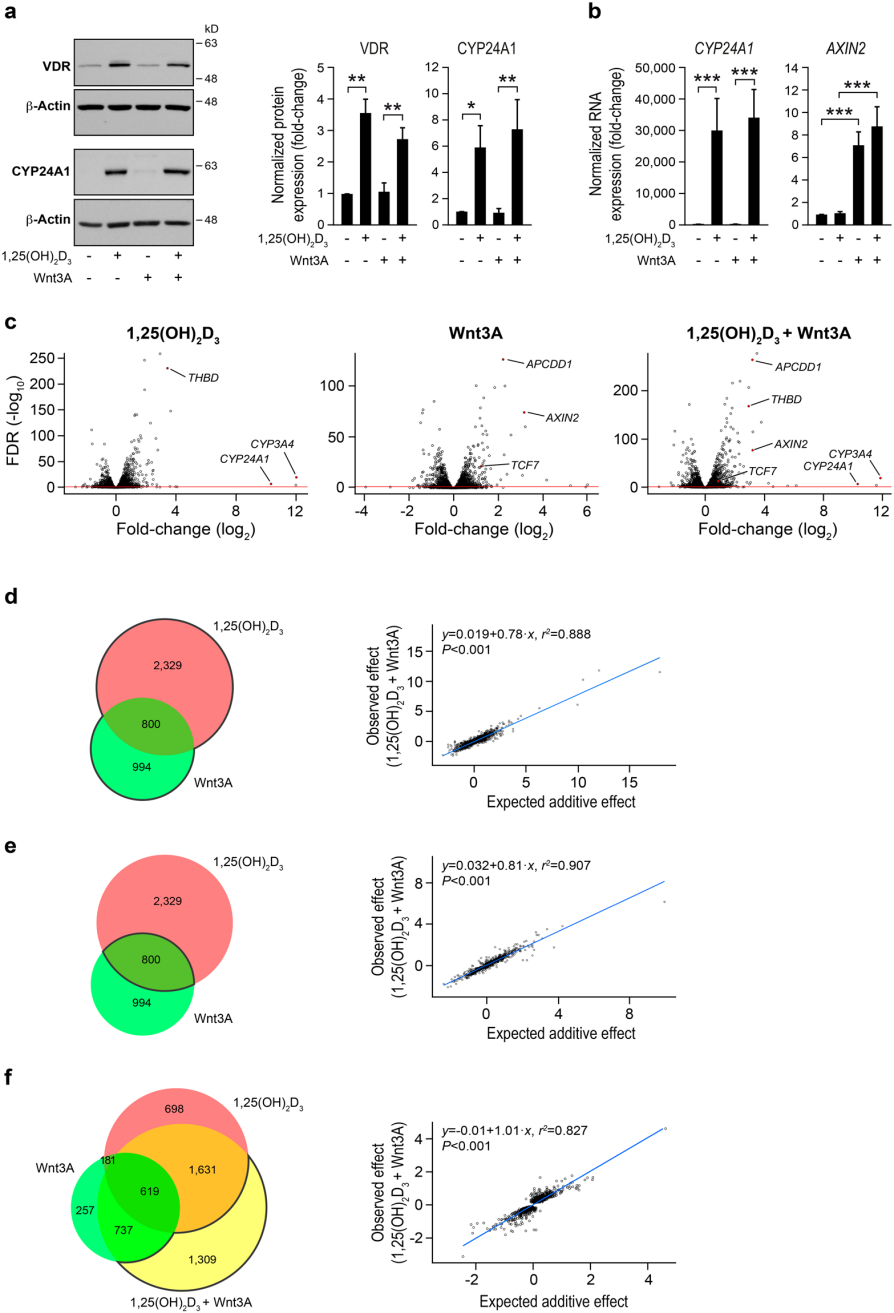
1,25(OH)_2_D_3_ and Wnt3A drastically regulate the gene expression program of CCD-18Co human colon myofibroblasts. (**a**) Western blot analysis of VDR and CYP24A1 protein levels in CCD-18Co cells treated with 1,25(OH)_2_D_3_ and/or Wnt3A for 24 h. β-Actin was used as a loading control. Images of a representative experiment (left) and the quantification (mean ± SEM) of three independent experiments (right) are shown. Full-length blots are presented in Supplementary Fig. S6. (**b**) RT-qPCR analysis of *CYP24A1* and *AXIN2* RNA levels in CCD-18Co cells treated as in (**a**). The mean ± SEM of three independent experiments is shown. (**c**) Volcano plots showing the RNA-seq results (FDR-adjusted *P* value *vs*. fold-change) obtained in CCD-18Co cells treated for 24 h with 1,25(OH)_2_D_3_ (left), Wnt3A (middle), or both (right) compared with vehicle-treated cells. Each dot represents a gene. Genes above the red line are considered differentially expressed (FDR-adjusted *P* value < 0.05). Some previously known target genes of 1,25(OH)_2_D_3_ (*THBD*, *CYP24A1*, and *CYP3A4*) or Wnt3A (*APCDD1*, *AXIN2*, and *TCF7*) are labelled. (**d**,**e**) Left, Venn diagram showing the overlap between the genes identified as significantly regulated by each single treatment (1,25(OH)_2_D_3_ or Wnt3A). The number of genes included in each group is depicted. Right, scattergram and simple linear regression analysis of the relationship between the observed effect of the combined treatment (log_2_ of the fold-change) and the expected additive effect (calculated by adding up the log_2_ of the fold-change of the single treatments) for the group of genes highlighted in the adjacent Venn diagram. Each dot represents a gene. (**f**) Left, Venn diagram showing the overlap among the genes identified as significantly regulated by the single (1,25(OH)_2_D_3_ or Wnt3A) or the combined (1,25(OH)_2_D_3_ + Wnt3A) treatment. The number of genes included in each group is depicted. Right, scattergram and simple linear regression analysis of the relationship between the observed effect of the combined treatment (log_2_ of the fold-change) and the expected additive effect (calculated by adding up the log_2_ of the fold-change of the single treatments) for the group of genes highlighted in the adjacent Venn diagram. Each dot represents a gene.

To examine the effects of 1,25(OH)_2_D_3_ and Wnt3A alone or in combination on colon fibroblasts, we chose the CCD-18Co human colon myofibroblast cell line. First, we sought to ensure CCD-18Co responsiveness to the two agents. CCD-18Co cells expressed a basal level of VDR protein that increased upon 1,25(OH)_2_D_3_ treatment (Fig. 1a). 1,25(OH)_2_D_3_ also enhanced the expression (RNA and protein) of *CYP24A1*, one of its known target genes that encodes the enzyme responsible for its degradation (Fig. 1a,b). Likewise, treatment of CCD-18Co cells with Wnt3A led to upregulation of its target gene *AXIN2* (Fig. 1b). These data showed that CCD-18Co myofibroblasts are responsive to 1,25(OH)_2_D_3_ and Wnt3A.

Next, we studied the effects of 24 h treatment with 1,25(OH)_2_D_3_ and Wnt3A, alone and in combination, on the gene expression profile of CCD-18Co cells by RNA-sequencing (RNA-seq) analysis. Three independent experiments were performed. Principal component analysis and a heatmap clustered by the Euclidean distance of raw RNA-seq data showed robust clustering of triplicates and strong separation among the different treatments (Supplementary Fig. S1). Interestingly, differential gene expression analysis of RNA-seq data indicated that a high number of genes were significantly (FDR-adjusted *P* value < 0.05) regulated by single treatment with 1,25(OH)_2_D_3_ or Wnt3A. 1,25(OH)_2_D_3_ changed the expression of 3,129 genes (51% induced and 49% repressed) in CCD-18Co cells, including some well-known 1,25(OH)_2_D_3_ target genes in other cell types (*THBD*, *CYP24A1*, and *CYP3A4*) (Fig. 1c and Supplementary Table S1), while Wnt3A modulated the expression of 1,794 genes (52% induced and 48% repressed), including its classic target genes *APCDD1*, *AXIN2*, and *TCF7* (Fig. 1c and Supplementary Table S2). A comparison of both groups of differentially expressed genes showed that 800 genes were common targets of 1,25(OH)_2_D_3_ and Wnt3A. A total of 55% (436/800) of them were induced (219/800) or repressed (217/800) by both single treatments. In contrast, the remaining 45% (364/800) of them were induced by the single treatment with 1,25(OH)_2_D_3_ and repressed by the single treatment with Wnt3A (228/800), or *vice versa* (136/800) (Fig. 1d, left panel and Supplementary Fig. S2).

An analysis of the combined treatment results and their comparison with the single treatment results provided interesting data. First, the number of genes regulated by the combination of 1,25(OH)_2_D_3_ + Wnt3A was higher (4,296 genes, 50% induced and 50% repressed) than those regulated by any of the single treatments (Fig. 1c and Supplementary Table S3). Second, a highly significant direct correlation existed between the observed experimental effect of the combined treatment (log_2_ of the fold-change *vs*. vehicle-treated cells) and the theoretically expected additive effect (calculated by the sum of the log_2_ of the fold-change *vs*. vehicle-treated cells of the single treatments) for all the genes differentially expressed by the single treatments (Fig. 1d), and specifically for the 800 genes that were common targets of the single treatments (Fig. 1e). In fact, the expected additive effect explained 89% (Fig. 1d, *r*^2^ = 0.888) and 91% (Fig. 1e, *r*^2^ = 0.907) of the variability observed in the combined treatment for the previously mentioned groups of genes. Third, 1,309 genes were significantly regulated by the combined treatment but not by any of the single treatments, and they also displayed a significant direct correlation between the observed effect of the combined treatment (log_2_ of the fold-change *vs*. vehicle-treated cells) and the expected additive effect (calculated by the sum of the log_2_ of the fold-change *vs*. vehicle-treated cells of the single treatments) (Fig. 1f). The expected additive effect explained 83% (*r*^2^ = 0.827) of the variability observed in the combined treatment (Fig. 1f), which suggests that most of these genes are also regulated by the single treatments, although the results did not achieve statistical significance. Altogether, these data indicated that the results of the combined treatment were largely predicted by the sum of the effects of the two single treatments.

**Figure 2:**
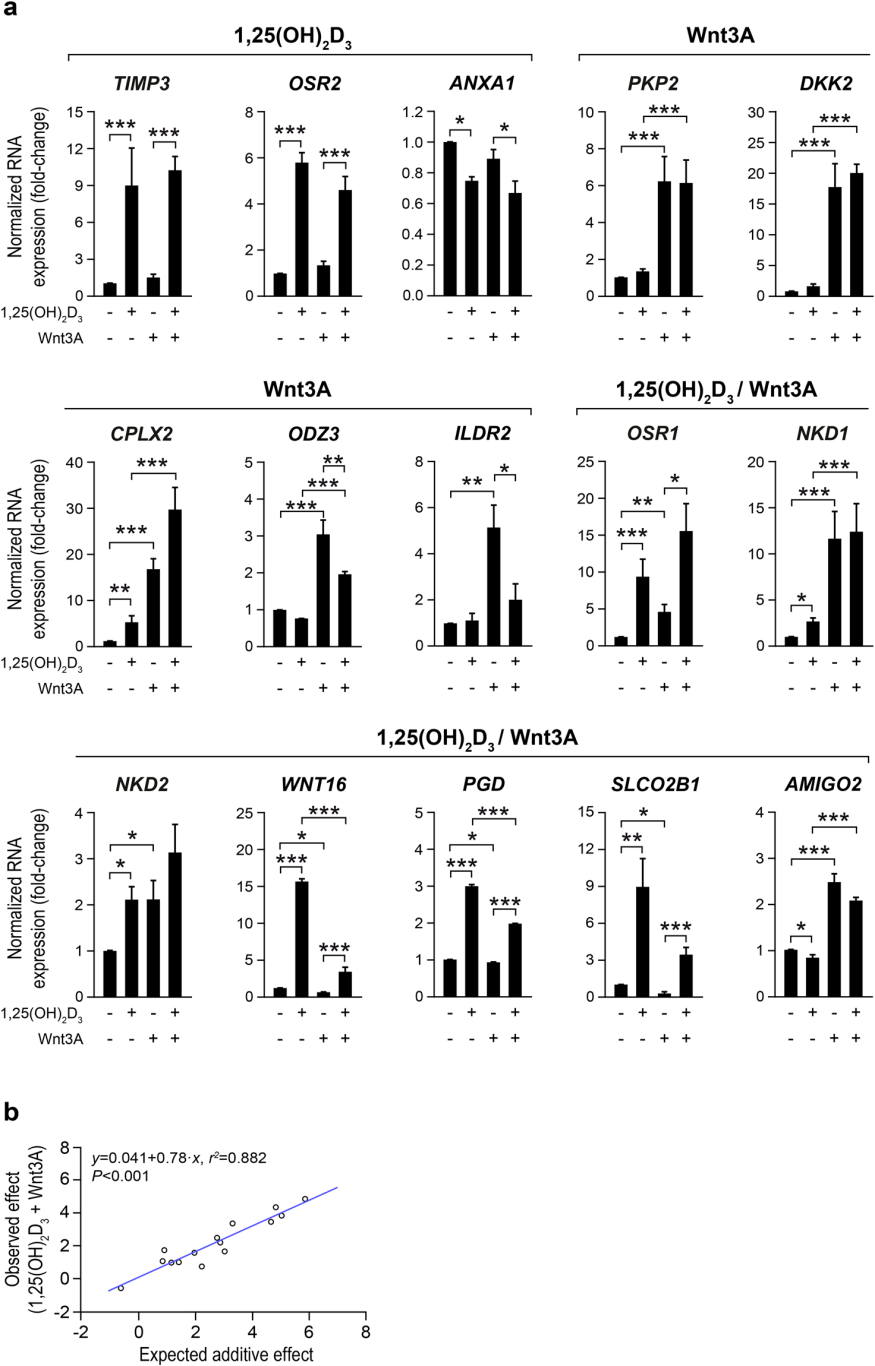
Validation by RT-qPCR of several genes identified as differentially expressed in the RNA-seq study. (**a**) RT-qPCR analysis of the RNA levels of the indicated genes in CCD-18Co cells treated with 1,25(OH)_2_D_3_ and/or Wnt3A for 24 h. Genes are grouped according to the single stimulus that regulates each gene in the RNA-seq data. The mean ± SEM of the fold-change *vs*. vehicle-treated cells in three independent experiments is depicted. (**b**) Scattergram and simple linear regression analysis of the relationship between the effect of the combined treatment (log_2_ of the fold-change *vs*. vehicle-treated cells) and the expected additive effect (calculated by adding up the log_2_ of the fold-change *vs*. vehicle-treated cells of the single treatments) for the genes analysed by RT-qPCR in (**a**). Each dot represents a gene.

In another three independent experiments, we validated by RT-qPCR the effects of 1,25(OH)_2_D_3_ and Wnt3A on the expression of a subset of genes identified as differentially expressed in the RNA-seq analysis: *TIMP3*, *OSR2*, and *ANXA1* were regulated by 1,25(OH)_2_D_3_; *PKP2*, *DKK2*, *CPLX2*, *ODZ3*, and *ILDR2* were regulated by Wnt3A; and *OSR1*, *NKD1*, *NKD2*, *WNT16*, *PGD*, *SLCO2B1*, and *AMIGO2* were regulated by both single treatments (Fig. 2a). In addition, the results showed a statistically significant induction of *CPLX2* by 1,25(OH)_2_D_3_, which was also detected in the RNA-seq analysis, although without statistical significance. The study also revealed an additive effect of 1,25(OH)_2_D_3_ and Wnt3A for most genes, except for *ILDR2*. Accordingly, a highly significant direct correlation was found in RT-qPCR results between the observed experimental effect of the combined treatment and the theoretically expected additive effect (Fig. 2b**)**. The expected additive effect explained 88% (*r*^2^ = 0.882) of the variability observed in the combined treatment (Fig. 2b).

### 1,25(OH)_2_D_3_ and Wnt3A modulate the phenotype of human colon myofibroblasts

**Figure 3:**
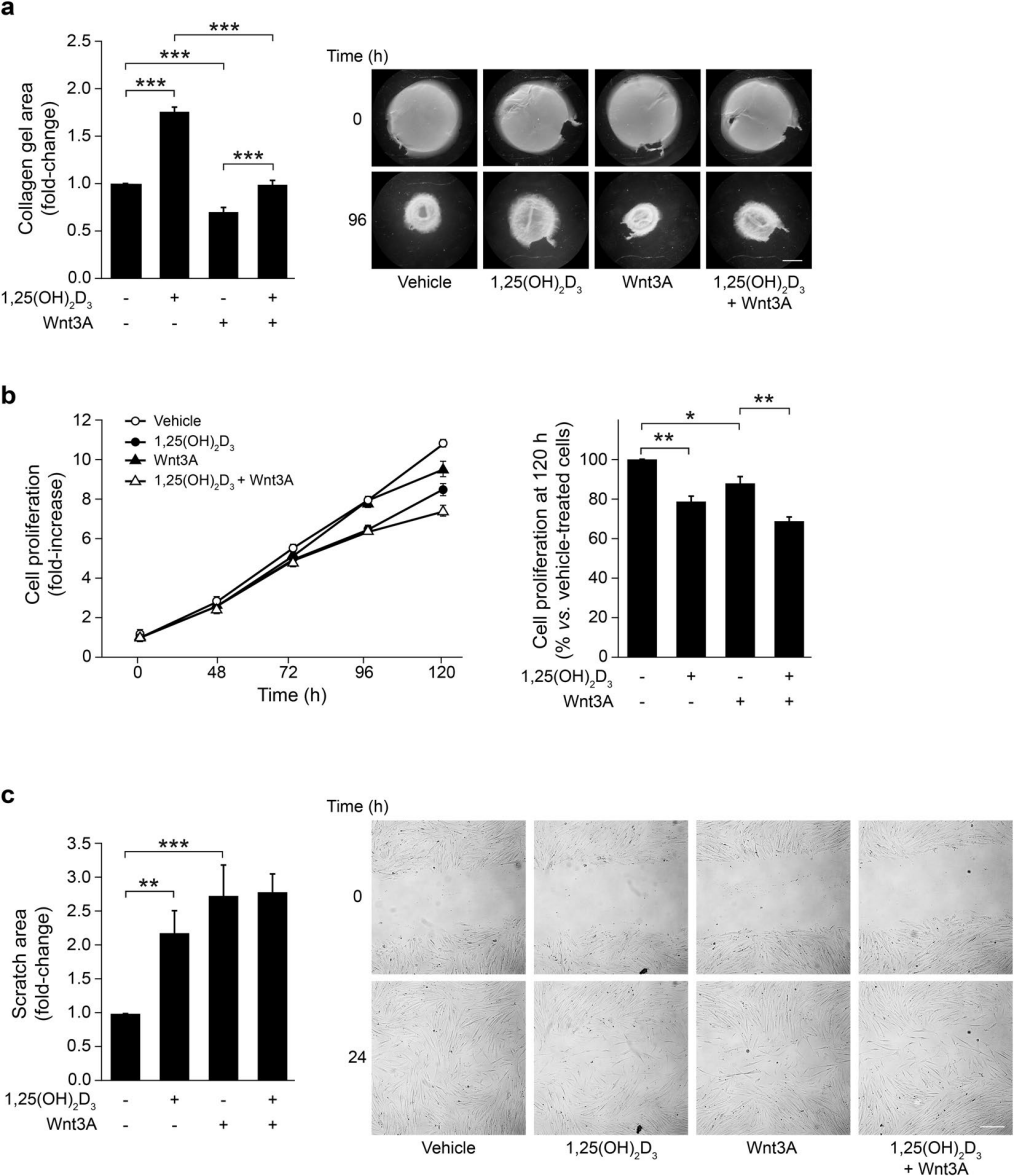
Effects of 1,25(OH)_2_D_3_ and Wnt3A on the phenotype of CCD-18Co human colon myofibroblasts. (**a**) Collagen gel contraction assay of CCD-18Co cells in the presence of 1,25(OH)_2_D_3_ and/or Wnt3A. The gel area was measured at 0 and 96 h. Results are shown as the fold-change in the gel area at 96 h calculated *vs*. vehicle-treated cells in three independent experiments (mean ± SEM) (left). Representative stereomicroscope images of collagen gels are depicted (right). Bar, 4 mm. (**b**) Proliferation of CCD-18Co cells treated with 1,25(OH)_2_D_3_ and/or Wnt3A. Left, cell proliferation was assessed at the indicated times and normalized *vs*. time 0 h (mean ± SEM of three independent experiments). Right, the graph depicts cell proliferation at 120 h calculated as percentage *vs*. vehicle-treated cells (mean ± SEM of three independent experiments). (**c**) Wound healing assay of CCD-18Co cells in the presence of 1,25(OH)_2_D_3_ and/or Wnt3A. The scratch area was measured at 0 and 24 h. Results are shown as the fold-change in the scratch area at 24 h calculated *vs*. vehicle-treated cells in three independent experiments (mean ± SEM) (left). Representative bright-field images of the scratches are depicted (right). Bar, 300 µm.

Next, we explored the effect of 1,25(OH)_2_D_3_ and Wnt3A on the phenotype of CCD-18Co cells. First, we studied the modulation by single or combined treatments of the ability of CCD-18Co cells to remodel the extracellular matrix, estimated as the capacity to contract collagen gels, which is a hallmark of activated fibroblasts. 1,25(OH)_2_D_3_ decreased the capacity of CCD-18Co cells to contract collagen gels, while Wnt3A had the opposite effect leading to a higher contraction capacity (Fig. 3a). The combined treatment significantly reduced the effect of each single agent, indicating that the actions of 1,25(OH)_2_D_3_ and Wnt3A balanced each other out and that both were equipotent, at least at the doses used (Fig. 3a). Supporting these data, 1,25(OH)_2_D_3_ inhibited the expression of *S100A4* in these cells, while Wnt3A induced that of *ACTA2*, which both are markers of activated fibroblasts (Supplementary Fig. S3).

Second, we studied the effects of 1,25(OH)_2_D_3_ and Wnt3A on CCD-18Co cell proliferation (Fig. 3b). At 120 h, the single treatment with 1,25(OH)_2_D_3_ or Wnt3A respectively caused a 21% or a 12% reduction in cell proliferation compared to vehicle-treated cells, while the combined treatment had an additive effect with 31% of inhibition respect to vehicle-treated cells (Fig. 3b, right panel).

Third, we explored the effect of 1,25(OH)_2_D_3_ and Wnt3A on the migration capacity of CCD-18Co cells measured by the wound healing assay. In this case, single treatment with 1,25(OH)_2_D_3_ or Wnt3A significantly decreased the ability of CCD-18Co cells to close the scratch area and the effect of the combined treatment was similar to that of the single treatments (Fig. 3c).

### Effects of 1,25(OH)_2_D_3_ and Wnt3A on patient-derived primary human colon fibroblasts

**Figure 4:**
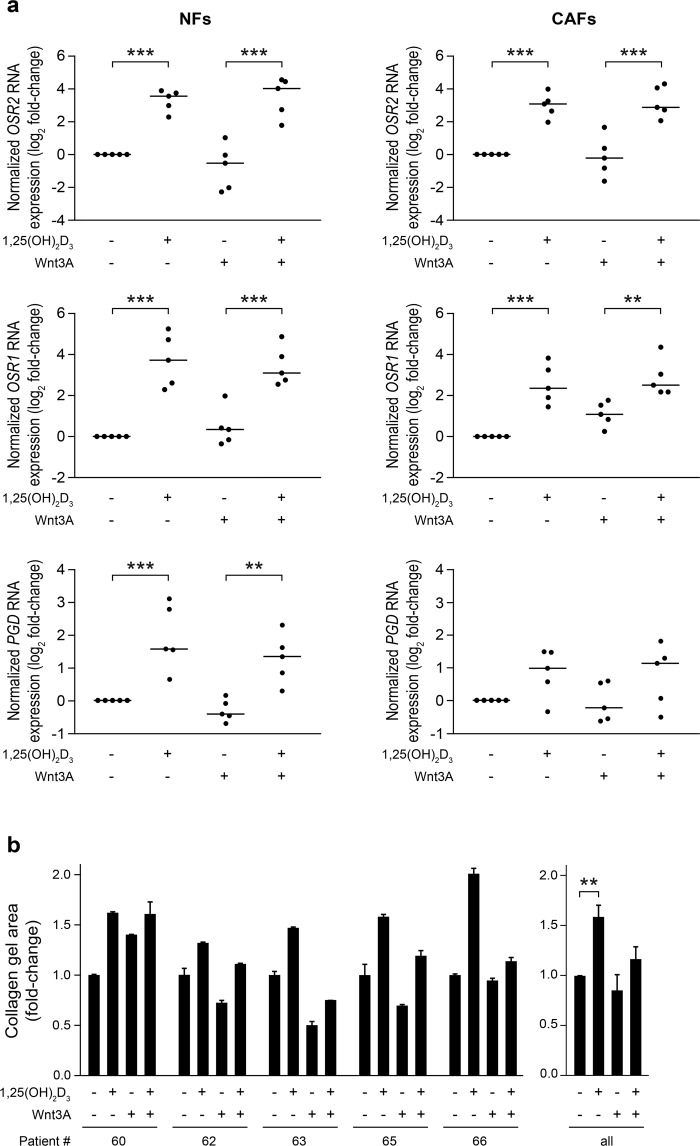
1,25(OH)_2_D_3_ and Wnt3A action in patient-derived primary human colon fibroblasts. (**a**) RT-qPCR analysis of the RNA expression of three genes identified in the RNA-seq study as significantly regulated by the single treatment with 1,25(OH)_2_D_3_ (*OSR2*, *OSR1*, and *PGD*) or Wnt3A (*OSR1* and *PGD*) was performed in five paired NF and CAF primary cultures derived from CRC patients (#60, #62, #63, #65, and #66) and treated with 1,25(OH)_2_D_3_ and/or Wnt3A for 24 h. Data are shown as log_2_ of the fold-change *vs*. vehicle-treated cells and the horizontal bars indicate the median values. (**b**) Collagen gel contraction assay of five primary cultures of NFs from the indicated patients in the presence of 1,25(OH)_2_D_3_ and/or Wnt3A. The gel area was measured at 0 and 96 h. Results are depicted as the fold-change in the gel area at 96 h calculated *vs*. vehicle-treated fibroblasts. Data from each patient (mean ± SD) (left) and the mean ± SEM of all patients (right) are shown.

To analyse whether the results obtained in CCD-18Co cells can be extended to other colon fibroblasts, we studied the effects of 1,25(OH)_2_D_3_ and Wnt3A in primary cultures of paired NFs and CAFs derived from colon healthy tissue or primary tumours, respectively, of CRC patients. First, we confirmed that NFs and CAFs respond to 1,25(OH)_2_D_3_ and Wnt3A with the induction of RNA expression of their respective target genes *CYP24A1* and *AXIN2* (Supplementary Fig. S4). Then, we analysed the RNA expression of three genes identified in the RNA-seq study as significantly regulated by the single treatment with 1,25(OH)_2_D_3_ (*OSR2*, *OSR1*, and *PGD*) or Wnt3A (*OSR1* and *PGD*) in five paired NFs and CAFs. Notably, the significant induction of the three genes by the single treatment with 1,25(OH)_2_D_3_ was reproduced in NFs and CAFs, except for *PGD* in CAFs that was induced by 1,25(OH)_2_D_3_ in four out of five primary cultures without reaching statistical significance (Fig. 4a). However, interindividual variability was found in the effect of the single treatment with Wnt3A: the induction of *OSR1* was observed in all the CAFs analysed but only in three out of five NFs, while the repression of *PGD* was detected in four out of five NFs and in three out of five CAFs (Fig. 4a). This variability precluded statistical significance and may be due to particular characteristics of the patients. In fact, we found a significant inverse correlation between the endogenous *AXIN2* RNA levels and their regulation by Wnt3A, and a similar tendency for *PGD* (Supplementary Fig. S5). In addition, as expected from the lack of a significant effect of the single treatment with Wnt3A on *OSR1* and *PGD* expression, the combined treatment did not significantly modify the effect of the single treatment with 1,25(OH)_2_D_3_ on the expression of these genes (for *OSR1*, *P* > 0.999 in NFs and *P* > 0.999 in CAFs; for *PGD*, *P* = 0.526 in NFs and *P* > 0.999 in CAFs) (Fig. 4a).

Moreover, we performed collagen gel contraction assays with five primary cultures of colon NFs. We found that the collagen gels treated with 1,25(OH)_2_D_3_ were larger than those treated with vehicle in all primary cultures analysed. Thus, 1,25(OH)_2_D_3_ significantly inhibited the capacity of NFs to contract collagen gels (Fig. 4b). In contrast, the effect of Wnt3A was variable: it increased the contraction of collagen gels by NFs from four patients (#62, #63, #65, and #66), while it had the opposite effect in the case of patient #60 (Fig. 4b). As occurred in CCD-18Co cells, the combined 1,25(OH)_2_D_3_ + Wnt3A treatment had an intermediate effect between both single treatments (Fig. 4b).

### 1,25(OH)_2_D_3_ and Wnt3A decrease the migration capacity of fibroblasts from different origins

**Figure 5:**
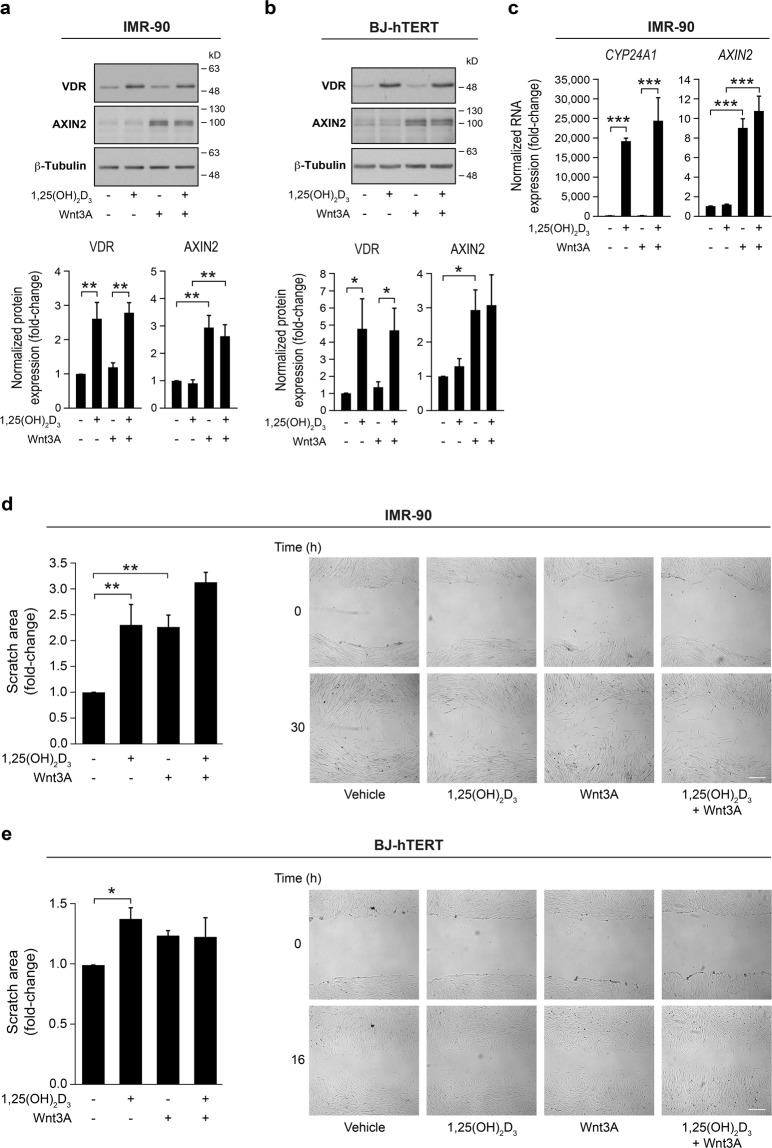
1,25(OH)_2_D_3_ and Wnt3A effects in IMR-90 human lung fibroblasts and BJ-hTERT human foreskin fibroblasts. (**a**,**b**) Western blot analysis of VDR and AXIN2 protein levels in IMR-90 (**a**) and BJ-hTERT (**b**) cells treated with 1,25(OH)_2_D_3_ and/or Wnt3A for 24 h. β-Tubulin was used as a loading control. Images of a representative experiment (upper panels) and the quantification (mean ± SEM) of three independent experiments (lower panels) are shown. Full-length blots are presented in Supplementary Figs. S7 and S8. (**c**) RT-qPCR analysis of *CYP24A1* and *AXIN2* RNA levels in IMR-90 fibroblasts treated as in (**a**). The mean ± SEM of three independent experiments is shown. (**d**,**e**) Wound healing assay of IMR-90 (**d**) and BJ-hTERT (**e**) fibroblasts in the presence of 1,25(OH)_2_D_3_ and/or Wnt3A. The scratch area was measured at 0 and 30 h (**d**) or at 0 and 16 h (**e**). Results are shown as the fold-change in the scratch area at 30 h (**d**) or at 16 h (**e**) calculated *vs*. vehicle-treated cells in three independent experiments (mean ± SEM) (left). Representative bright-field images of the scratches are depicted (right). Bar, 300 µm.

To extend our results to fibroblasts from other tissues, we analysed the response to 1,25(OH)_2_D_3_ and Wnt3A of IMR-90 human lung fibroblasts and BJ-hTERT human foreskin fibroblasts. Both types of fibroblasts expressed VDR and responded to 1,25(OH)_2_D_3_ and Wnt3A (Fig. 5a–c). Similarly to the effect observed in CCD-18Co cells, single treatment with 1,25(OH)_2_D_3_ or Wnt3A significantly reduced the migration of IMR-90 fibroblasts, while the combined treatment displayed a slightly higher inhibitory effect (Fig. 5d). In the case of BJ-hTERT fibroblasts, 1,25(OH)_2_D_3_ also significantly reduced cell migration while Wnt3A showed a tendency that did not reach statistical significance (*P* = 0.073) (Fig. 5e).

## Discussion

Stromal fibroblasts are crucial in healthy and pathologic intestine. However, little is known about the signals that regulate their biology. Here we have analysed the effect on colon fibroblasts of canonical Wnt factors present in the intestinal stem cell niche and in the CRC tumour microenvironment. In addition, we studied the action of 1,25(OH)_2_D_3_, an important regulator of gut physiology that inhibits the Wnt/β-catenin signalling pathway in colon carcinoma cells^24–26,31^.

Our results show that single treatment with 1,25(OH)_2_D_3_ or Wnt3A has wide effects on gene expression and phenotype in human colon fibroblasts. In both cases, approximately half of the differentially expressed genes are upregulated, probably a subset of them by direct binding of VDR or TCF to their regulatory regions (such as the classic target genes highlighted in Fig. 1c), and others indirectly in cascade by the products of the direct target genes. Regarding the downregulated genes, it is widely accepted that the mechanisms responsible for gene repression by 1,25(OH)_2_D_3_/VDR and Wnt/β-catenin are mostly indirect and mediated by the products of direct target genes, miRNAs, or by the interaction of VDR or β-catenin with transcription factors or signalling pathways^32,33^. The number of differentially expressed genes (3,129 *vs*. 1,794) and the magnitude of the regulation (maximum fold-change 4,166 *vs*. 64) reveal that 1,25(OH)_2_D_3_ is a more potent regulator of fibroblast gene expression than Wnt3A, at least under the experimental conditions used.

We analysed the effects of 1,25(OH)_2_D_3_ and Wnt3A on several fibroblast properties that have been associated with an activated, protumoural phenotype (extracellular matrix contraction, proliferation, and migration)^19^. Our data clearly indicate that 1,25(OH)_2_D_3_ inhibits these properties in several types of human fibroblasts, including patient-derived primary colon fibroblasts. This is in line with results obtained in other systems^27,34–37^ and supports the fact that 1,25(OH)_2_D_3_ is a master modulator of activated stroma with promising therapeutic applications in cancer and fibrotic diseases. However, the data obtained with Wnt3A are more complex, as it increases the fibroblast capacity to contract collagen gels and the expression of the classic myofibroblast marker *ACTA2*, while it reduces fibroblast proliferation and migration. These findings suggest that the diverse fibroblast properties analysed here are regulated by different intracellular mechanisms and target genes. Accordingly, although activation of the Wnt/β-catenin signalling pathway in fibroblasts usually promotes the acquisition of a myofibroblast phenotype associated with enhanced proliferation and migration^38–41^, some exceptions reveal that these cellular properties are not always coupled and, indeed, are sometimes modulated in opposite ways by the activation of the Wnt/β-catenin pathway^42–46^. Thus, it has been reported that Wnt3A treatment or overexpression of a constitutively active β-catenin mutant inhibits cell proliferation in mouse embryonic fibroblasts^42,44^. Likewise, Wnt3A reduces cell proliferation in melanocytes and in several melanoma and B-cell acute lymphoblastic leukaemia cell lines^47–49^. Similarly, the canonical Wnt1 protein inhibits human umbilical vein endothelial cell proliferation^50^. In addition, stimulation with Wnt3A reduces the invasive capacity of mouse mesenchymal stem cells, while the inhibition of the Wnt/β-catenin signalling pathway by β-catenin knockdown exerts the opposite effect^51^. Also, Stojadinovic *et al*. showed that β-catenin stabilization inhibits keratinocyte migration during wound healing^52^. Interestingly, mammary fibroblasts engineered to secrete Wnt3A have been found to promote or inhibit tumour growth in xenografts derived from two different breast cancer patients^53^. Moreover, Kabiri *et al*. have recently described that inhibition of Wnt signalling enhances proliferation of intestinal stem cells^54^. These variable effects of the activation of the Wnt/β-catenin signalling pathway are probably due to the specific molecular characteristics of the different cellular systems and highlight the cell type- and context-dependent effects of Wnt signals.

A comparison of the differentially expressed genes shows that most genes regulated by the single treatment with 1,25(OH)_2_D_3_ (74%; 2,329/3,129) or Wnt3A (55%; 994/1,794) are unshared, while 800 genes are common targets. This suggests that the gene regulatory action of both agents is mainly independent and differentiated. Remarkably, 55% (436/800) of the commonly regulated genes were induced or repressed by both single treatments, while the remaining 45% (364/800) were induced by one of the single treatments but repressed by the other one. Consistently, common and opposite effects are exerted by 1,25(OH)_2_D_3_ and Wnt3A on the CCD-18Co cell phenotype: both agents inhibit CCD-18Co cell proliferation and migration, while 1,25(OH)_2_D_3_ reduces, but Wnt3A induces, their contraction capacity.

Interestingly, the results obtained with the combined 1,25(OH)_2_D_3_ + Wnt3A treatment reveal a predominantly additive effect on CCD-18Co gene expression. First, a detailed analysis of RNA-seq data indicates that the sum of the effects of the single treatments explains 89% (*r*^2^ = 0.888) of the variability observed in the combined treatment for the genes that are differentially expressed by the single treatments. A similar result was found for the genes studied by RT-qPCR. Secondly, 70% (2,987/4,296) of the genes that are differentially expressed by the combined treatment are also significantly regulated by any of the single treatments. And third, the sum of the effects of the single treatments also explains 83% (*r*^2^ = 0.827) of the variability observed in the combined treatment for the 1,309 genes that are differentially expressed only by 1,25(OH)_2_D_3_ + Wnt3A, which suggests that those genes are also regulated by the single treatments, although the results did not achieve statistical significance. Accordingly, both agents display a clearly additive effect on the contraction capacity of CCD-18Co and patient-derived primary colon fibroblasts, CCD-18Co cell proliferation, and IMR-90 cell migration.

Previous studies by our group and others have shown that 1,25(OH)_2_D_3_ inhibits the Wnt/β-catenin signalling pathway in colon carcinoma cells mainly through the induction of VDR binding to β-catenin, which leads to the disruption of TCF/β-catenin complexes, and by the upregulation of E-cadherin that promotes β-catenin relocation from the nucleus to the cell membrane adherens junctions^24,55^. Additionally, the Wnt/β-catenin pathway is inhibited by 1,25(OH)_2_D_3_ in colon carcinoma cells in a paracrine manner: 1,25(OH)_2_D_3_ diminishes the secretion by macrophages of interleukin-1β, which acts on colon carcinoma cells and stabilizes β-catenin^56^. In contrast to a general inhibitory effect, our results in human colon fibroblasts reveal that 1,25(OH)_2_D_3_ does not globally change the expression of Wnt3A target genes, although it significantly reduces the effect of Wnt3A on 23% (410/1,794) of the genes regulated by Wnt3A alone, including the known Wnt target genes *CCND1*, *DKK1*, *MMP14*, *TNFRSF19*, and *CYR61*. Analogously, Meyer *et al*. found that 1,25(OH)_2_D_3_ only modestly influences β-catenin binding sites on the genome of LS180 colon carcinoma cells^57^, which suggests that 1,25(OH)_2_D_3_ may neither generally affect the expression of β-catenin target genes in other cell types. Additional data support a role of 1,25(OH)_2_D_3_ as a partial inhibitor of the Wnt/β-catenin signalling pathway in colon fibroblasts: (i) a 1,25(OH)_2_D_3_-induced VDR/β-catenin interaction has been observed in CCD-18Co fibroblasts^58^; and (ii) our RNA-seq results show that certain Wnt inhibitors (*NKD1*, *NKD2* and *APCDD1*) are induced by 1,25(OH)_2_D_3_.

In summary, our findings reinforce the relevance of 1,25(OH)_2_D_3_ and Wnt3A in healthy and pathologic intestine, and indicate that in addition to their action on intestinal normal epithelial cells and on colon carcinoma cells, they are strong regulators of colon fibroblast biology. Thus, their actions on intestinal stroma should be considered for a better understanding of intestinal homeostasis and for the design of therapies against CRC and other intestinal diseases.

## Methods

### Cells and cell culture

CCD-18Co (ATCC CRL-1459) human colon myofibroblasts were cultured in Minimum Essential Medium plus 10% foetal bovine serum (FBS) (both from Life Technologies). IMR-90 (ATCC CCL-186) human lung fibroblasts and BJ-hTERT (ATCC CRL-4001) human foreskin fibroblasts immortalized with hTERT were cultured in Dulbecco’s modified Eagle’s medium (Life Technologies) plus 10% FBS. Cell lines were periodically authenticated using the GenePrint 10 System (Promega) and the results were sent for comparison against the ATCC cell line database. Lyophilised recombinant human Wnt3A (#5036-WN, R&D Systems) was reconstituted at a concentration of 200 μg/ml in PBS containing 0.1% BSA following manufacturer’s indications. Cells were treated with a final concentration of 100 nM 1,25(OH)_2_D_3_ (Sigma-Aldrich), 100 ng/ml recombinant human Wnt3A, and/or with the corresponding volume of vehicles (ethanol for 1,25(OH)_2_D_3_; 0.1% BSA in PBS for Wnt3A) for the indicated times (in long experiments cells were retreated every 48 h).

### Primary cultures of human colon fibroblasts

Primary cultures of human colon normal and tumour fibroblasts were established using the explant outgrowth technique as previously described^27,59,60^. Briefly, fresh surgical specimens from morphologically normal colon mucosa (at least 5 cm from the surgical margin) and from colon primary tumour of the same patient were obtained after surgery and incubated with antibiotics and fungicides. Then, samples were cut into small pieces and placed in T25 cell culture flasks with FBS plus 0.25 mg/ml Primocin (InvivoGen). After one week, FBS was replaced by Fibroblast Growth Medium-2 (FGM-2, Lonza). Fibroblasts grew around the tissue explants for approximately 3 weeks. Then, tissue fragments were removed and fibroblasts were routinely cultured in FGM-2. All experiments were performed with primary fibroblasts at seventh passage at most. Human samples were obtained with the approval of the Ethics Committee for Clinical Research of Hospital Universitario La Paz (approval code: HULP-PI-1425) and provided by the Biobank of the Instituto de Investigación Sanitaria Hospital Universitario La Paz (IdiPAZ Biobank, PT13/0010/0003, Plataforma de Apoyo a la Investigación en Ciencias y Tecnologías de la Salud en la Red de Biobancos 2013). The research with human samples was performed in accordance with the Declaration of Helsinki, and Spanish and EU legislation. All patients gave written informed consent.

### RNA-sequencing and bioinformatics analyses

Total RNA from three independent experiments of CCD-18Co human colon myofibroblasts treated with 1,25(OH)_2_D_3_, Wnt3A, both, or vehicle for 24 h was extracted using RNeasy Mini Kit (Qiagen). Sequencing libraries were prepared with the Illumina TruSeq Stranded mRNA Sample Preparation Kit as indicated in the Illumina TruSeq Stranded mRNA Sample Preparation Guide (#15031047 D). Briefly, polyA + fractions were purified and randomly fragmented, converted to double stranded cDNA and processed through subsequent enzymatic treatments of end-repair, dA-tailing, and ligation to adapters. Adapter-ligated library was completed by 9 cycles of PCR with Illumina PE primers. The resulting purified cDNA libraries were sequenced (single-read, 40-bases read length) on an Illumina Genome Analyzer IIx at the Genomics Facility of the Spanish National Cancer Research Centre using Illumina TruSeq SBS Kit v5 and following manufacturer’s protocols. Image analysis and per-cycle base calling were performed with Illumina Real-Time Analysis (RTA1.13) software. Conversion to fastq read format and sequence alignment to the GRCh37/hg19 assembly of the human genome with ELAND v2e alignment algorithm were achieved using Illumina CASAVA 1.8 software. The raw reads (fastq files) and the raw number of reads *per* gene are available at the Gene Expression Omnibus database (http://www.ncbi.nlm.nih.gov/gds/) under the accession number GSE120106.

The normalization of the raw number of reads *per* gene and the differential gene expression analysis were performed with the Bioconductor package DESeq2^61^ designed for R statistical computing software (https://www.r-project.org/). To account for multiple hypotheses testing, the estimated significance level (*P* value) was adjusted using Benjamini & Hochberg False Discovery Rate (FDR) correction. Those genes with a FDR-adjusted *P* value < 0.05 were selected as differentially expressed by 1,25(OH)_2_D_3_, Wnt3A or 1,25(OH)_2_D_3_ + Wnt3A treatment *vs*. vehicle-treated cells. Venn diagrams were performed with BioVenn software (http://www.biovenn.nl/)^62^ or VennDiagram (https://CRAN.R-project.org/package = VennDiagram) designed for R statistical computing software.

### RNA isolation and quantitative RT-PCR

Total RNA was extracted using RNeasy Mini Kit (Qiagen). RNA was retrotranscribed using iScript cDNA Synthesis kit (Bio-Rad). Then, the quantitative PCR (qPCR) reaction was performed in a CFX384 Real-Time PCR detection System (Bio-Rad) using TaqMan Universal Master Mix II (Life Technologies). Thermal cycling of the qPCR reaction was initiated with a denaturation step at 95 °C for 10 min and consisted of 40 cycles (denaturation at 95 °C for 15 s, annealing and elongation at 60 °C for 30 s). We used the following TaqMan probes: *CYP24A1* (Hs00167999_m1), *AXIN2* (Hs00610344_m1), *TIMP3* (Hs00927214_m1), *OSR2* (Hs00369588_m1), *ANXA1* (Hs00167549_m1), *PKP2* (Hs00428040_m1), *DKK2* (Hs 00205294_m1), *CPLX2* (Hs00932617_m1), *ODZ3* (Hs01111780_m1), *ILDR2* (Hs01025498_m1), *OSR1* (Hs04189871_m1), *NKD1* (Hs00263894_m1), *NKD2* (Hs01108239_m1), *WNT16* (Hs00365138_m1), *PGD* (Hs00427230_m1), *SLCO2B1* (Hs01030343_m1), *AMIGO2* (Hs00827141_g1), *S100A4* (Hs00243202_m1), *ACTA2* (Hs00426835_g1), *RPLP0* (Hs99999902_m1), *GAPDH* (Hs02758991_g1), and *B2M* (Hs99999907_m1) (all from Life Technologies). For RNA expression of cell lines (CCD-18Co, IMR-90 and BJ-hTERT), RNA expression values were normalized *vs*. the housekeeping gene *RPLP0* using the comparative C_T_ method. For RNA expression of patient-derived fibroblasts, RNA expression values were independently normalized *vs*. three housekeeping genes (*RPLP0*, *GAPDH*, and *B2M*) using the comparative C_T_ method and the mean was calculated. All experiments were performed using triplicates.

### Western blot

Whole-cell extracts were prepared by cell lysis with RIPA buffer plus protease- and phosphatase-inhibitors for 25 min on ice, followed by centrifugation at 13,000 rpm for 10 min at 4 °C. Proteins were separated by SDS-PAGE, transferred to PVDF membranes and incubated with antibodies against VDR (#12550, Cell Signaling Technology), CYP24A1 (sc-66851, Santa Cruz Biotechnology), AXIN2 (#2151, Cell Signaling Technology), β-actin (sc-1616, Santa Cruz Biotechnology), and β-tubulin (T4026, Sigma-Aldrich), and then with HRP-conjugated secondary antibodies. Antibody binding was visualized using the ECL detection system (GE Healthcare). Films were photographed with a Nikon Coolpix S700 camera and images were processed using Adobe Photoshop CC software. Quantification was done by densitometry using ImageJ software (National Institutes of Health). Protein expression values were first normalized *vs*. the housekeeping protein β-actin (for CCD-18Co fibroblasts) or β-tubulin (for IMR-90 and BJ-hTERT fibroblasts), and then *vs*. vehicle-treated fibroblasts.

### Cell proliferation

The cell proliferation assay used is based on the cleavage of the yellow [3-(4,5-dimethythiazol-2-yl)-2,5-diphenyl] tetrazolium bromide salt (MTT) to purple formazan crystals by metabolic active cells. CCD-18Co fibroblasts were seeded in 24-well cell culture plates and treated with 1,25(OH)_2_D_3_, Wnt3A, both, or vehicle. At the indicated times, cells were incubated with the MTT solution (final concentration of 0.5 mg/ml, Merck Millipore) for 4 h at 37 °C. After this incubation period, a water-insoluble formazan dye is formed. After solubilisation in isopropanol 0.04 M HCl during 30 min at RT, the formazan dye was quantified using a VersaMax scanning microplate spectrophotometer (Molecular Devices). The absorbance was measured as 570–630 nm. All experiments were performed using triplicates.

### Collagen gel contraction assay

Collagen gels were prepared by mixing fibroblasts with PureCol bovine type I collagen (Advanced Biomatrix), 5x DMEM, 0.1 M NaOH, and distilled water (final concentrations were 1.7 mg/ml PureCol, 1x DMEM, and 3 mM NaOH) in the presence of 1,25(OH)_2_D_3_, Wnt3A, both, or vehicle. The mixture was seeded in 24-well cell culture plates and allowed to polymerize for 1 h at 37 °C. Then, culture medium with the corresponding treatments (1,25(OH)_2_D_3_, Wnt3A, both, or vehicle) was added. After 24 h and to initiate gel contraction (time 0), gels were gently released from the 24-well plates and transferred into 6-well plates containing culture medium with 1,25(OH)_2_D_3_, Wnt3A, both, or vehicle. Gels were photographed at 0 and 96 h with a Leica DFC550 digital camera mounted in a Leica S6D stereomicroscope and the gel areas were measured with ImageJ software. Images were processed using Adobe Photoshop CC software. All experiments were performed using triplicates.

### Wound healing assay

Fibroblasts were seeded in 6-well cell culture dishes. Almost (around 80%) confluent fibroblasts were treated O/N with 1,25(OH)_2_D_3_, Wnt3A, both, or vehicle. Then, wounds were created by lightly scratching a straight-line across the cell monolayers with a 200 µl plastic pipette tip. After 2 washes with PBS to remove detached cells, medium supplemented with 0.5% FBS plus the indicated treatment (1,25(OH)_2_D_3_, Wnt3A, both, or vehicle) was added. Cells were incubated in an inverted Cell Observer Z1 microscope (Zeiss) coupled to an incubation system with regulated CO_2_ and temperature and images at different time intervals were captured with a Cascade 1 K camera (Photometrics) using AxioVision Rel. 4.8 software (Zeiss). The scratch areas at the indicated time points were quantified by ImageJ software and images were processed using Adobe Photoshop CC software. All experiments were performed using triplicates.

### Statistical analyses

Results are expressed as mean ± SEM unless otherwise specified. Statistical significance was assessed by unpaired one-way ANOVA analysis with Bonferroni multiple comparison post-test using GraphPad Prism 7 software. The following comparisons were assessed: vehicle *vs*. 1,25(OH)_2_D_3_, vehicle *vs*. Wnt3A, 1,25(OH)_2_D_3_ *vs*. 1,25(OH)_2_D_3_ + Wnt3A, and Wnt3A *vs*. 1,25(OH)_2_D_3_ + Wnt3A. Differences were considered significant when *P* < 0.05. The single asterisk indicates *P* < 0.05, the double asterisk *P* < 0.01, and the triple asterisk *P* < 0.001. GraphPad Prism 7 software or R statistical computing software were used to calculate the simple linear regression and the Pearson correlation coefficient of the relationships between the observed effect of the combined treatment (log_2_ of the fold-change of 1,25(OH)_2_D_3_ + Wnt3A- *vs*. vehicle-treated cells) and the expected additive effect (log_2_ of the fold-change of 1,25(OH)_2_D_3_- *vs*. vehicle-treated cells plus log_2_ of the fold-change of Wnt3A- *vs*. vehicle-treated cells), and between RNA expression in vehicle-treated (log_2_ of the fold-change *vs*. vehicle-treated NFs from patient #60; endogenous levels) and in Wnt3A-treated (log_2_ of the fold-change *vs*. the corresponding vehicle-treated fibroblasts; Wnt3A-regulated levels) patient-derived primary human colon fibroblasts.

## Supplementary information

Supplementary Information

Supplementary Dataset Table S1

Supplementary Dataset Table S2

Supplementary Dataset Table S3

## Data Availability

The RNA-seq data generated during the current study are available at the Gene Expression Omnibus database (http://www.ncbi.nlm.nih.gov/gds/) under the accession number GSE120106.
