# Supplementary material for: Vitamin D and Wnt3A have additive and partially overlapping modulatory effects on gene expression and phenotype in human colon fibroblasts
Source: Sci Rep. 2019 May 30;9:8085. doi: 10.1038/s41598-019-44574-9 (PMC6542853; doi:10.1038/s41598-019-44574-9)
Supplement: Supplementary file 1 — Supplementary Information [file 41598_2019_44574_MOESM1_ESM.pdf]

Vitamin D and Wnt3A have additive and partially overlapping modulatory effects on gene expression and phenotype in human colon fibroblasts

Gemma Ferrer-Mayorga[1,2,3], Núria Niell[1,4,^], Ramón Cantero[2,5], José Manuel González-Sancho[1,3,4], Luis del Peso[1,2,4,6], Alberto Muñoz[1,2,3,*] & María Jesús Larriba[1,2,3,*]

[1]Instituto de Investigaciones Biomédicas "Alberto Sols", Consejo Superior de Investigaciones Científicas, Universidad Autónoma de Madrid, Madrid, Spain.

[2]Instituto de Investigación Sanitaria Hospital Universitario La Paz, Madrid, Spain.

[3]CIBERONC, Instituto de Salud Carlos III, Madrid, Spain.

[4]Departamento de Bioquímica, Facultad de Medicina, Universidad Autónoma de Madrid, Madrid, Spain.

[5]Servicio de Cirugía General, Hospital Universitario La Paz, Madrid, Spain.

[6]CIBERES, Instituto de Salud Carlos III, Madrid, Spain.

[^]Present address: Departamento de Biología, Facultad de Ciencias, Universidad Autónoma de Madrid, Madrid, Spain.

[*]Corresponding authors.

Correspondence and requests for materials should be addressed to A.M. (email: amunoz@iib.uam.es) or M.J.L. (email: mjlarriba@iib.uam.es).

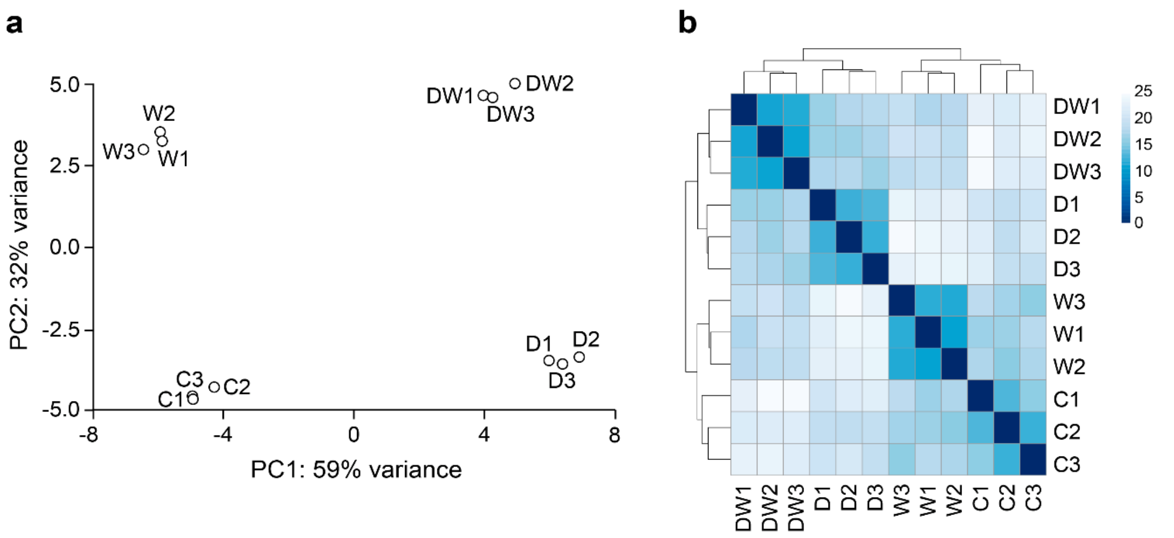

Supplementary Figure S1. Analysis of raw RNA-seq data reveals clustering of replicates and strong separation among samples belonging to different treatments. (**a**) Principal component analysis of raw RNA-seq data from CCD-18Co cells treated with vehicle (C), 1,25(OH)$_2$D$_3$ (D), Wnt3A (W), or both (DW) in three independent experiments (1-2-3). (**b**) Heatmap clustered by the Euclidean distance of CCD-18Co cells treated with vehicle (C), 1,25(OH)$_2$D$_3$ (D), Wnt3A (W), or both (DW) in three independent experiments (1-2-3) based on raw RNA-seq data.

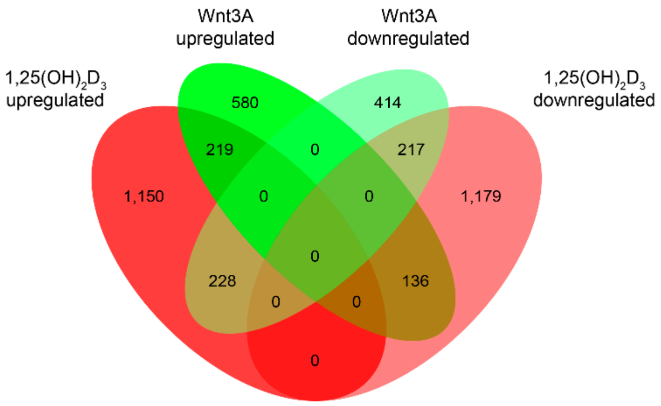

Supplementary Figure S2. Comparison of genes regulated by single treatment with $1,25(OH)_2D_3$ or Wnt3A in CCD-18Co human colon myofibroblasts. Venn diagram showing the overlap between the genes identified in the RNA-seq study as significantly regulated by each single treatment ($1,25(OH)_2D_3$ or Wnt3A) and divided into upregulated and downregulated. The number of genes included in each group is depicted.

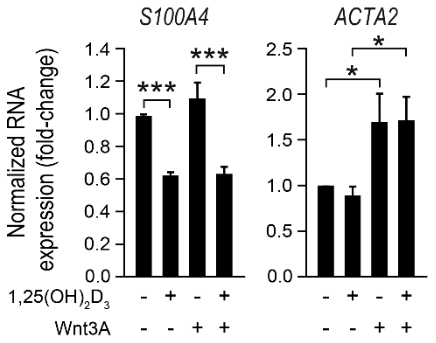

Supplementary Figure S3. 1,25(OH)$_2$D$_3$ inhibits and Wnt3A induces the expression of markers of activated fibroblasts in CCD-18Co human colon myofibroblasts. RT-qPCR analysis of *S100A4* and *ACTA2* RNA levels in CCD-18Co cells treated with 1,25(OH)$_2$D$_3$ and/or Wnt3A for 24 h. The mean ± SEM of the fold-change *vs*. vehicle-treated cells in three independent experiments is depicted.

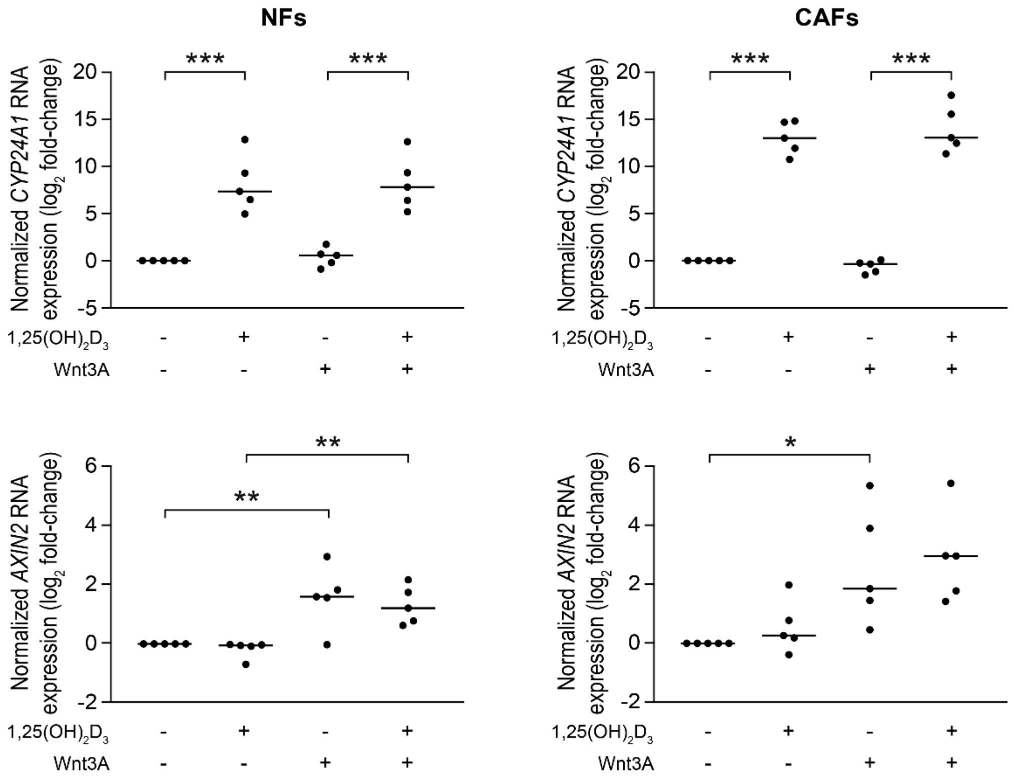

Supplementary Figure S4. Patient-derived primary human colon normal and tumour fibroblasts respond to 1,25(OH)$_2$D$_3$ and Wnt3A. RT-qPCR analysis of *CYP24A1* and *AXIN2* RNA levels in five paired NF and CAF primary cultures derived from CRC patients (#60, #62, #63, #65, and #66) and treated with 1,25(OH)$_2$D$_3$ and/or Wnt3A for 24 h. Data are shown as log$_2$ of the fold-change *vs*. vehicle-treated cells and the horizontal bars indicate the median values.

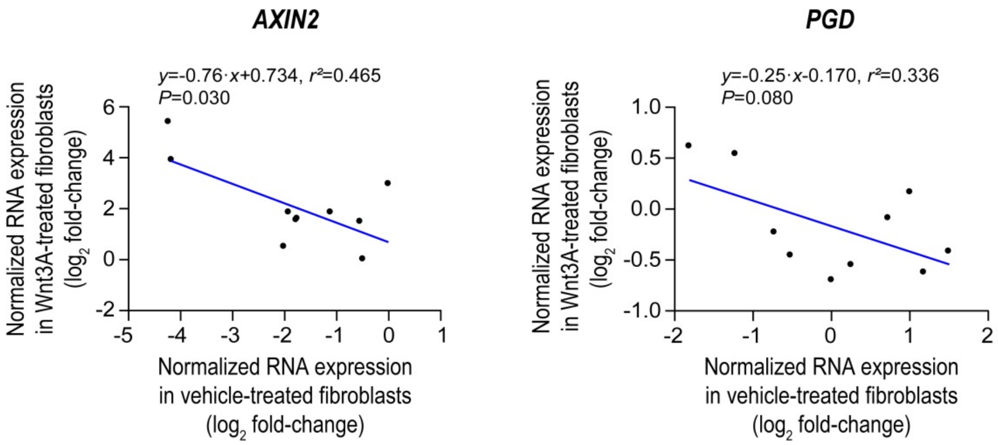

Supplementary Figure S5. The endogenous *AXIN2* and *PGD* RNA levels of patient-derived primary human colon normal and tumour fibroblasts inversely correlate with their regulation by Wnt3A. RT-qPCR analysis of *AXIN2* and *PGD* RNA levels in five paired NF and CAF primary cultures derived from CRC patients (#60, #62, #63, #65, and #66) and treated with Wnt3A or vehicle for 24 h. Scattergrams and simple linear regression analyses of the relationship between *AXIN2* or *PGD* RNA expression in vehicle-treated (log_2 of the fold-change *vs.* vehicle-treated NFs from patient #60; endogenous levels) and in Wnt3A-treated (log_2 of the fold-change *vs.* the corresponding vehicle-treated fibroblasts; Wnt3A-regulated levels) fibroblasts.

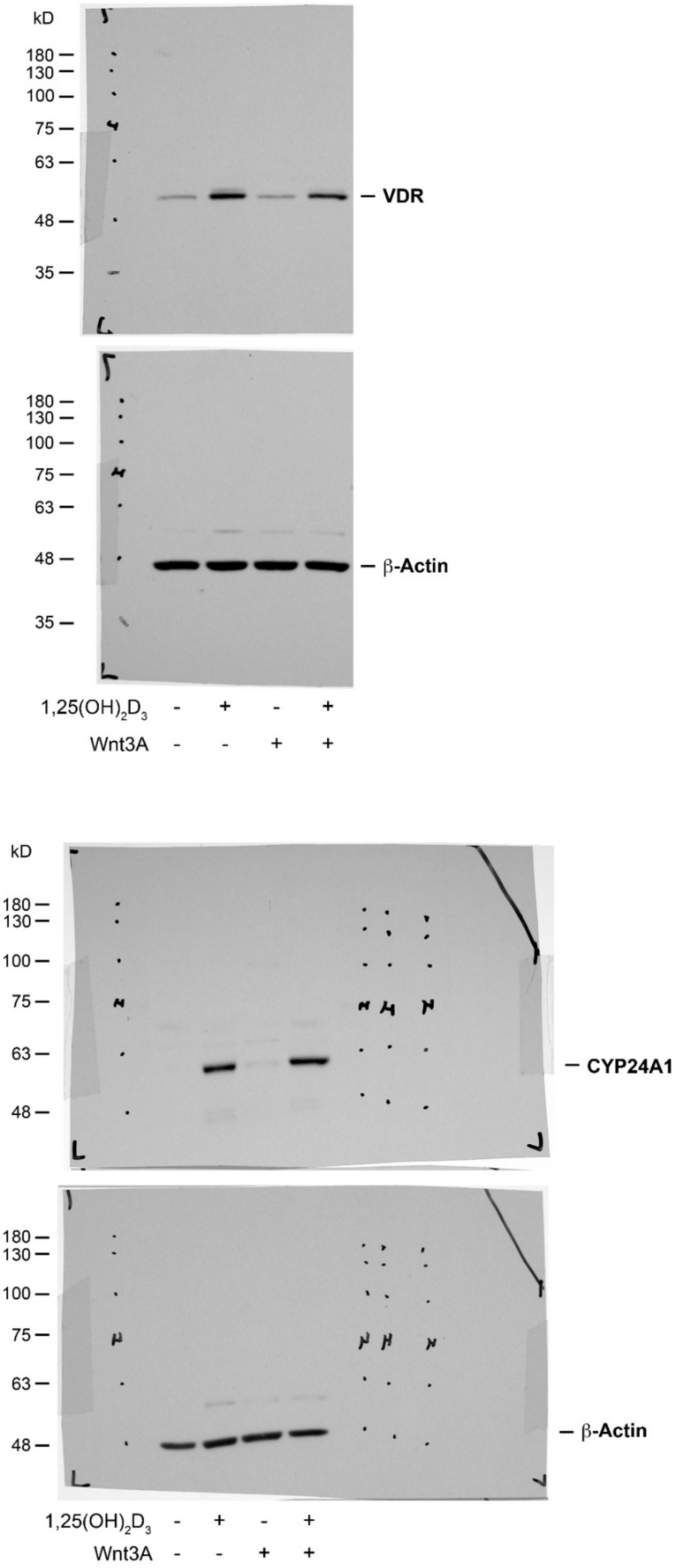

Supplementary Figure S6. Original uncropped full-length images of the western blots shown in Fig. 1a.

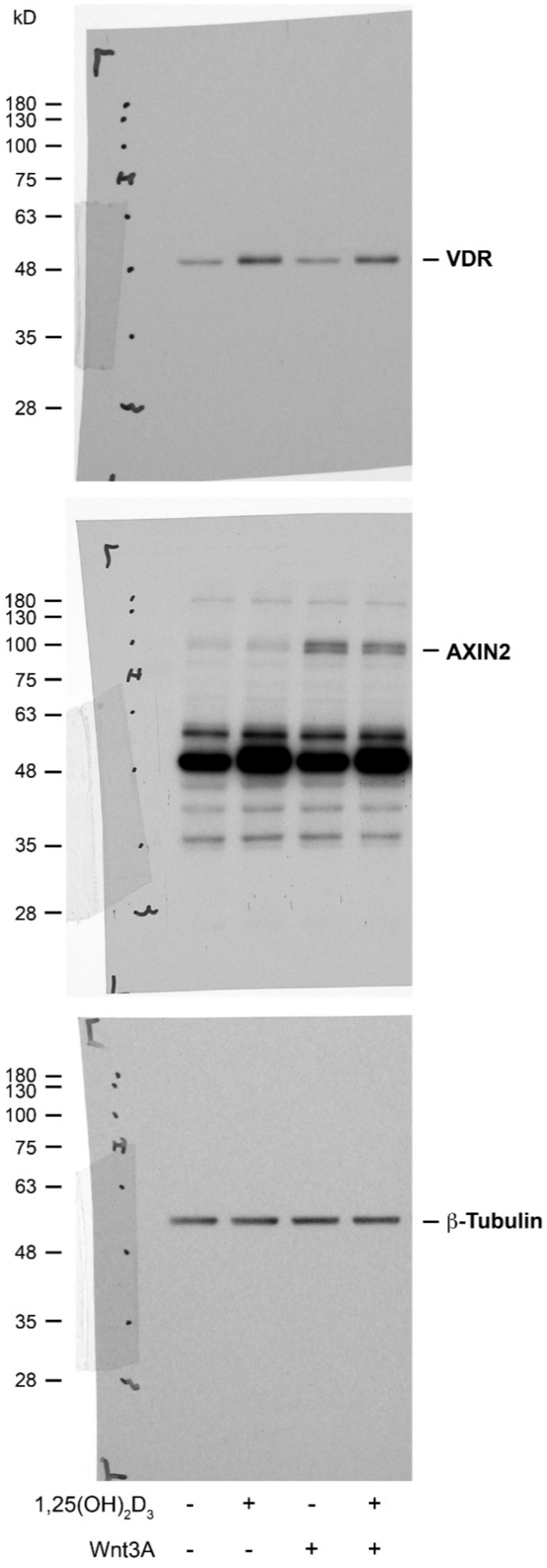

Supplementary Figure S7. Original uncropped full-length images of the western blots shown in Fig. 5a.

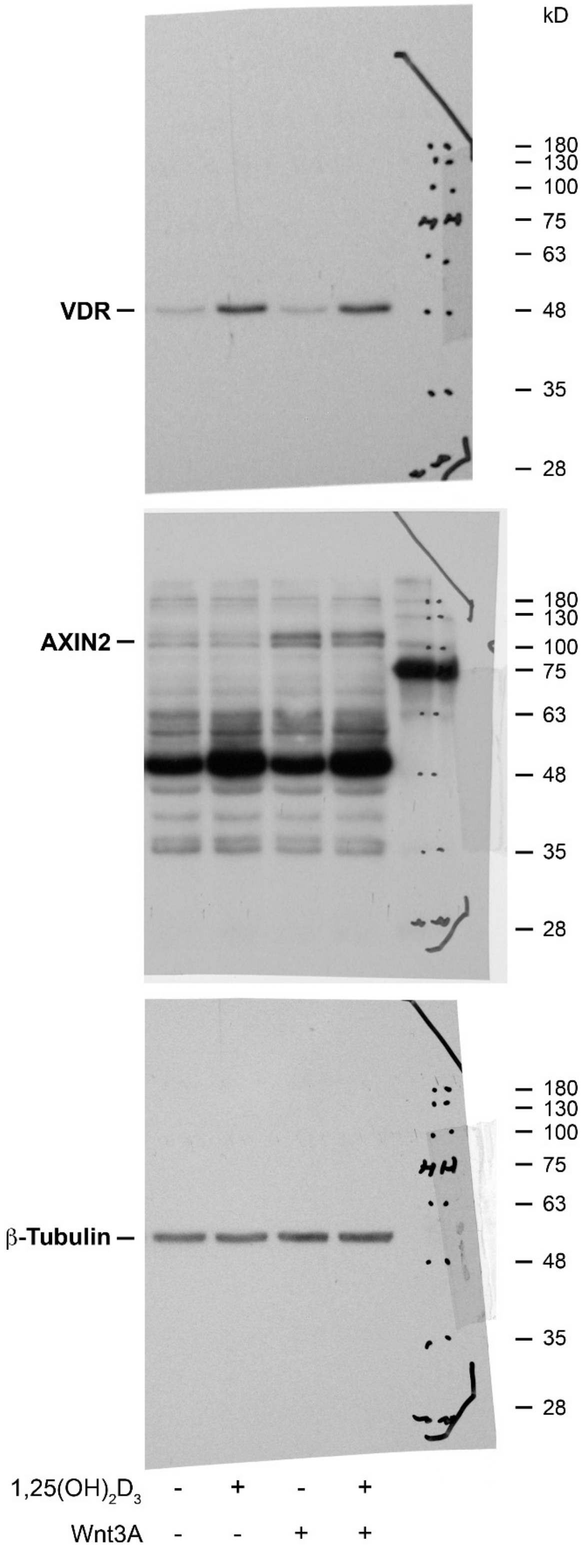

Supplementary Figure S8. Original uncropped full-length images of the western blots shown in Fig. 5b.
